# An Adaptive Bluetooth/Wi-Fi Fingerprint Positioning Method based on Gaussian Process Regression and Relative Distance

**DOI:** 10.3390/s19122784

**Published:** 2019-06-21

**Authors:** Hongji Cao, Yunjia Wang, Jingxue Bi, Hongxia Qi

**Affiliations:** 1 Key Laboratory of Land Environment and Disaster Monitoring, MNR, China University of Mining and Technology, Xuzhou 221116, China; hjcao@cumt.edu.cn; 2 School of Environmental Science and Spatial Informatics, China University of Mining and Technology, Xuzhou 221116, China; bjx1050@163.com (J.B.); hongxiaqi@yeah.net (H.Q.)

**Keywords:** Bluetooth, Wi-Fi, Gaussian process regression, relative distance, fingerprint positioning

## Abstract

Trusted positioning data are very important for the fusion of Bluetooth fingerprint positioning (BFP) and Wi-Fi fingerprint positioning (WFP). This paper proposes an adaptive Bluetooth/Wi-Fi fingerprint positioning method based on Gaussian process regression (GPR) and relative distance (RD), which can choose trusted positioning results for fusion. In the offline stage, measurements of the Bluetooth and Wi-Fi received signal strength (RSS) were collected to construct Bluetooth and Wi-Fi fingerprint databases, respectively. Then, fingerprint positioning error prediction models were built with GPR and data from the fingerprint databases. In the online stage, online Bluetooth and Wi-Fi RSS readings were matched with the fingerprint databases to get a Bluetooth fingerprint positioning result (BFPR) and a Wi-Fi fingerprint positioning result (WFPR). Then, with the help of RD and fingerprint positioning error prediction models, whether the positioning results are trusted was determined. The trusted result is selected as the position estimation result when there is only one trusted positioning result among the BFPR and WFPR. The mean is chosen as the position estimation result when both the BFPR and WFPR results are trusted or untrusted. Experimental results showed that the proposed method was better than BFP and WFP, with a mean positioning error of 2.06 m and a root-mean-square error of 1.449 m.

## 1. Introduction

Indoor positioning technology has attracted extensive attention as an important part of location-based services (LBS). In recent years, researchers have proposed some indoor positioning methods based on different technologies such as ultra-wideband (UWB) [1], Bluetooth [2,3], wireless fidelity (Wi-Fi) [4,5], Radio Frequency Identification (RFID) [6], computer vision [7], ultrasonic [8], infrared [9], inertial navigation system (INS) [10], etc. Among them, Wi-Fi positioning [11] technology can use existing Wi-Fi devices. Its positioning methods include those based on received signal strength (RSS) [12] and channel state information (CSI) [13]. A CSI-based positioning method requires special equipment, and it is thus difficult to popularize. However, an RSS-based positioning method can realize positioning on mobile platforms such as smartphones. Bluetooth has a small size and convenient installation, along with the same positioning theory as Wi-Fi. Its use range increases every year, and it has become one of the more important means of communication. Therefore, with the popularization and application of Bluetooth and Wi-Fi, positioning methods based on these two technologies will likely become universal for indoor positioning.

Fingerprint positioning [14,15] is easy to implement and simple in structure, and is a common method for Bluetooth and Wi-Fi positioning. Fingerprint positioning includes offline and online stages: the aim of the offline stage is fingerprint acquisition and fingerprint database construction [16], and that of the online phase is positioning by matching online RSS readings with the fingerprint database. There have been many studies on Wi-Fi fingerprint positioning (WFP) and Bluetooth fingerprint positioning (BFP), but studies on the fusion of BFP and WFP have been relatively few. For example, Bluetooth and Wi-Fi fingerprints have been used for indoor positioning in previous research [17,18,19,20]. However, both Wi-Fi and Bluetooth signals exist in certain indoor environments such as office buildings, supermarkets, etc. Thus, it is necessary to study the fusion of Bluetooth and Wi-Fi positioning technologies to make the most of existing signals for indoor positioning. 

A few studies have investigated fusion positioning using Wi-Fi and Bluetooth; for example, a study [21] used the path loss model to realize the fusion of Bluetooth and Wi-Fi distance positioning, but they did not study the fusion positioning method based on Bluetooth and Wi-Fi fingerprints. Su [22] mixed Bluetooth and Wi-Fi fingerprints to achieve positioning, comparing it with the WFP. However, they not only lacked a comparison with BFP, but also did not study the fusion of BFP and WFP. De Blasio [23] realized the fusion of BFP and WFP by using Wi-Fi for rough positioning and Bluetooth low energy (BLE) for fine positioning, but Wi-Fi positioning with a large error would offer an unreliable hunting zone, which can make the error of the BLE positioning large. The above methods cannot identify a trusted positioning result between the Wi-Fi and Bluetooth positioning results. Positioning results with a large error used in the fusion could make the error of the fusion result large. Thus, it is important to determine that the positioning results used in the fusion of Wi-Fi and Bluetooth positioning are trusted. Gaussian process regression (GPR) could establish the relationship between RSSs and their underlying positions [24]. GPR could also infer the posterior received signal strength (RSS) mean and RSS variance at each fingerprint location, and the posterior mean and variance of RSS data are utilized in fingerprint positioning [25,26]. Thus, we propose to utilize GPR to model the relationship between RSSs and their positioning error in this work.

This paper proposes an adaptive Bluetooth/Wi-Fi fingerprint positioning method (ABWFP), based on GPR and relative distance (RD), which can determine whether the Bluetooth fingerprint positioning result (BFPR) and Wi-Fi fingerprint positioning result (WFPR) are trusted. It realizes the adaptive mixing of BFP and WFP and improves the positioning accuracy and stability. In the offline stage, both Bluetooth and Wi-Fi RSS measurements are collected; these are then used to produce the Bluetooth and Wi-Fi reference fingerprints by taking the RSS mean values. The reference fingerprints and known coordinates constitute Bluetooth reference points (RPs) and Wi-Fi RPs, which are used to construct the Bluetooth and Wi-Fi fingerprint databases. Then, the reference fingerprints and their positioning errors are trained by GPR for the fingerprint positioning error prediction model. In the online positioning stage, the Bluetooth and Wi-Fi RSS readings are collected at the same time and are matched with the Bluetooth and Wi-Fi fingerprint databases, respectively. Then, RD is used to determine whether there is an abnormal positioning result, and the two positioning results are considered to be trusted if the RD between BFPR and WFPR is small. When the RD between BFPR and WFPR is large, it indicates that there is an abnormal positioning result. The online RSS readings are input into the fingerprint positioning error prediction models to predict the errors of BFPR and WFPR. Then, whether the fingerprint positioning results are trusted is determined by the prediction errors. If only the WFPR is trusted, it is selected as the position estimation result. Likewise, if only the BFPR is trusted, it is chosen as the positioning estimation result. If both positioning results are trusted, the mean of WFPR and BFPR should be taken as the positioning estimation result. If neither of the two positioning results is credible, the mean is still taken as the positioning result.

## 2. Training Data

Generally speaking, a fingerprint database is only used for matching positioning, and few studies have used the data in the fingerprint database to evaluate the positioning result. However, fingerprint acquisition requires a great deal of manpower and material resources, and subsequent update and maintenance will also consume substantial resources. Thus, it is a huge waste of resources that the fingerprint database is only used for positioning. 

Therefore, this paper proposes to use the data in the fingerprint database to build the fingerprint positioning error prediction model, which establishes the mapping relationship between fingerprints and positioning errors. In the process of real-time positioning, the prediction error can be obtained by using the online RSS readings and the fingerprint positioning error prediction model.

**Figure 1:**
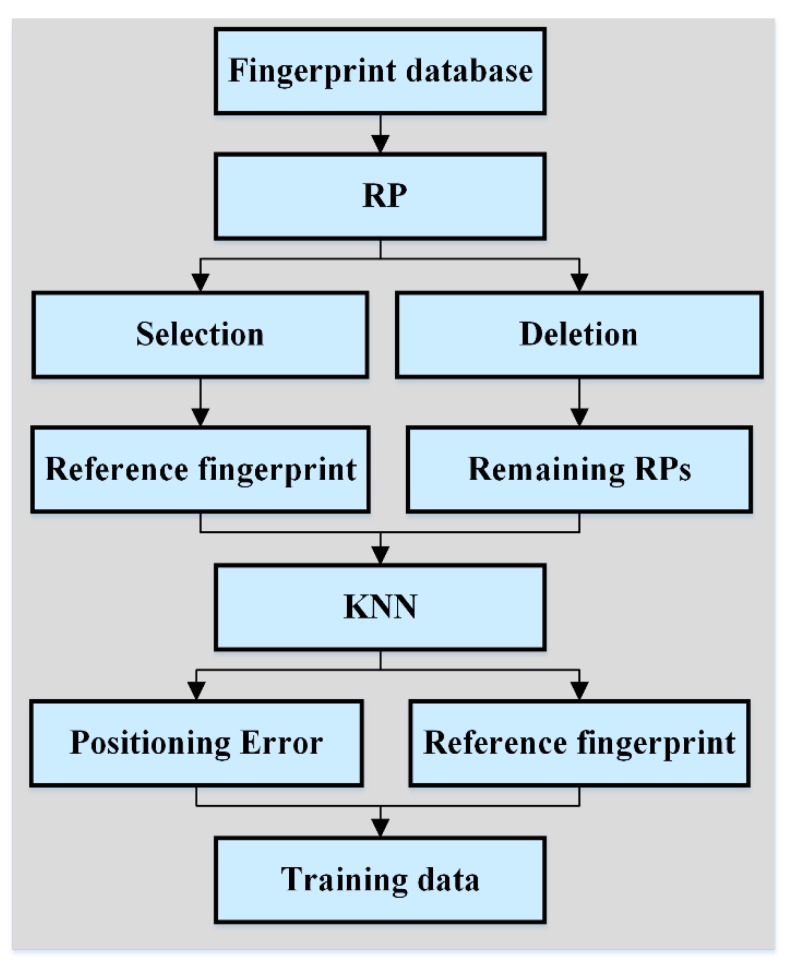
Flow chart of the process of acquiring training data.

For GPR training, training data must be first obtained. This section describes in detail the method for obtaining such data. As can be seen in Figure 1, an RP was firstly selected, and then the fingerprint database deleted this RP. The reference fingerprint of the RP was matched with the remaining RPs for fingerprint positioning. The *k*-nearest neighbor (KNN) algorithm is the positioning algorithm used in this work. The positioning result of the reference fingerprint was gained and used to calculate the positioning errors with the coordinates of the RP. The reference fingerprint and its corresponding positioning error make up the training data for the training positioning error prediction model. When all reference fingerprints of the RPs are used for matching positioning, the positioning errors corresponding to all reference fingerprints can be obtained. The number of training data is determined by the number of RPs.

## 3. The GPR-Based Fingerprint Positioning Prediction Model

### 3.1. Gaussian Process Regression

A Gaussian process is a set of random variables that are subject to a joint Gaussian distribution that is determined by a mean function and covariance function, as shown in Equation (1):

(1) $$f\left(x\right)∼GP\left(m\left(x\right),k\left(x_{i},x_{j}\right)\right)$$

where m(x) is the mean function that can be computed with Equation (2), $k\left(x_{i},y_{j}\right)$ is the covariance function that can be calculated with Equation (3), and f(x) represents the Gaussian process.

(2) m(x)=E[f(x)]

(3) $${k(x}_{i}{,x}_{j})=E\left[\left(f\left(x_{i}\right)-m\left(x_{i}\right)\right)\left(f\left(x_{j}\right)-m\left(x_{j}\right)\right)\right]$$

Here, E indicates the expectation operator. The kernel function in this work is expressed in Equation (4). $\sigma_{f}$ and l are the hyper-parameters, $\sigma_{f}$ is the signal standard deviation, and l is the length-scale parameter. This paper chose the Euclidean distance to calculate $k\left(x_{i},x_{j}\right)$, which is represented by $∥x_{i}-x_{j}∥$.

(4) $$k\left(x_{i},x_{j}\right)=\sigma_{f}^{2}\exp\left(-\frac{∥x_{i}-x_{j}∥}{2l^{2}}\right)$$

The covariance matrix is shown in Equation (5), where *N* is the number of training data.

(5) $$K=\left[\begin{array}{cccc} k\left(x_{1},x_{1}\right) & k\left(x_{1},x_{2}\right) & \cdots & k\left(x_{1},x_{N}\right)\\ k\left(x_{2},x_{1}\right) & k\left(x_{2},x_{2}\right) & \cdots & k\left(x_{2},x_{N}\right)\\ \vdots & \vdots & \ddots & \vdots \\ k\left(x_{N},x_{1}\right) & k\left(x_{N},x_{2}\right) & \cdots & k\left(x_{N},x_{N}\right) \end{array}\right]$$

The prediction positioning errors $y_{*}$ and training positioning errors y follow a multivariate Gaussian distribution jointly as follows:

(6) $$\left[\begin{array}{c} y\\ y_{*} \end{array}\right]∼N\left(\begin{array}{cc} 0, & \left[\begin{array}{cc} K\left(X,X\right) & K\left(X,X_{*}\right)\\ K\left(X_{*},X\right) & K\left(X_{*},X_{*}\right) \end{array}\right] \end{array}\right)$$

where $X_{*}$ and X are the test data and reference fingerprints, respectively. The posterior distribution $p\left(y_{*}|y\right)$ can be expressed as

(7) $$y_{*}|y∼N\left(K\left(X_{*},X\right)K{\left(X,X\right)}^{-1}y,K\left(X_{*},X_{*}\right)-K\left(X_{*},X\right)K{\left(X,X\right)}^{-1}K\left(X,X_{*}\right)\right)$$

Therefore, the posterior positioning error at each positioning result can be computed from the online RSS readings. However, the hyper-parameters should be calculated before estimating the positioning error.

### 3.2. GPR Hyper-Parameter Estimation

Solution of the hyper-parameters is the precondition of GPR. The aim of GPR training is to maximize the log-likelihood function. The log-likelihood function can be expressed as

(8) $$logp\left(y_{*}|y,\theta \right)=-\frac{1}{2}y^{T}K^{-1}y-\frac{1}{2}\log\left|K\right|-\frac{N}{2}\log2\pi$$

where θ represents the hyper-parameters [$\sigma_{f}$,l]. The Quasi-Newton method is a classic optimization method which can solve nonlinear optimization problems. Thus, we used the Broyden–Fletcher–Goldfarb–Shanno (BFGS) algorithm to compute the hyper-parameters.

The BFGS algorithm is an effective quasi-Newton method. It uses the gradient of the objective function to calculate the hyper-parameters. The gradient can be expressed as

(9) $$Q\left(\theta \right)=\frac{\partial \log\left(y_{*}|y,\theta \right)}{\partial \theta }$$

In order to calculate the hyper-parameters, the initial hyper-parameters $\theta_{0}$ and initial iteration matrix $B_{0}$ must be chosen for iteration. The θ of the (*k +* 1*)*th iteration can be calculated using Equation (10), where the step length $\lambda_{k}$ is determined by Wolfe conditions.

(10) $$\theta_{k+1}=\theta_{k}-\lambda_{k}B_{k}^{-1}Q_{k}$$

The iteration matrix $B_{k+1}$ of the (*k +* 1)th iteration is calculated using Equation (11), where $\delta_{k}$ = $\theta_{k+1}$−$\theta_{k}$ and $q_{k}$ = $Q\left(\theta_{k+1}\right)$−$Q\left(\theta_{k}\right)$. The iteration will be terminated if the absolute value $\left|q\left(\theta_{k}\right)\right|$ satisfies the termination condition.

(11) $$B_{k+1}=B_{k}-\frac{B_{k}\delta_{k}\delta_{{}_{k}}^{T}B_{k}}{\delta_{{}_{k}}^{T}B_{k}\delta_{k}}+\frac{q_{k}q_{k}^{T}}{q_{k}^{T}\delta_{k}}$$

The hyper-parameters were acquired using the BFGS algorithm. The resulting hyper-parameters for the Wi-Fi training data were (1.9048, 21.793), and the hyper-parameters for the Bluetooth training data were (0.6386, 9.8015).

## 4. Proposed Positioning Method

### 4.1. Adaptive Fingerprint Positioning Method for Bluetooth and Wi-Fi using GPR and RD

Under ideal conditions, BFPR and WFPR should be similar when BFR and WFP are used for positioning at the same time. However, the two positioning results may differ greatly in reality due to various reasons, such as multipaths, diffraction, scattering, etc. Thus, we used the RD between BFPR and WFPR to determine whether there is an abnormal positioning result.

A small RD of BFPR and WFPR indicates that BFPR and WFPR are trusted. When the RD between BFPR and WFPR is very large, this indicates the existence of an abnormal positioning result, which may be either BFPR or WFPR, but cannot help us to identify which positioning result is abnormal. However, the GPR-based fingerprint positioning error prediction model can do that.

**Figure 2:**
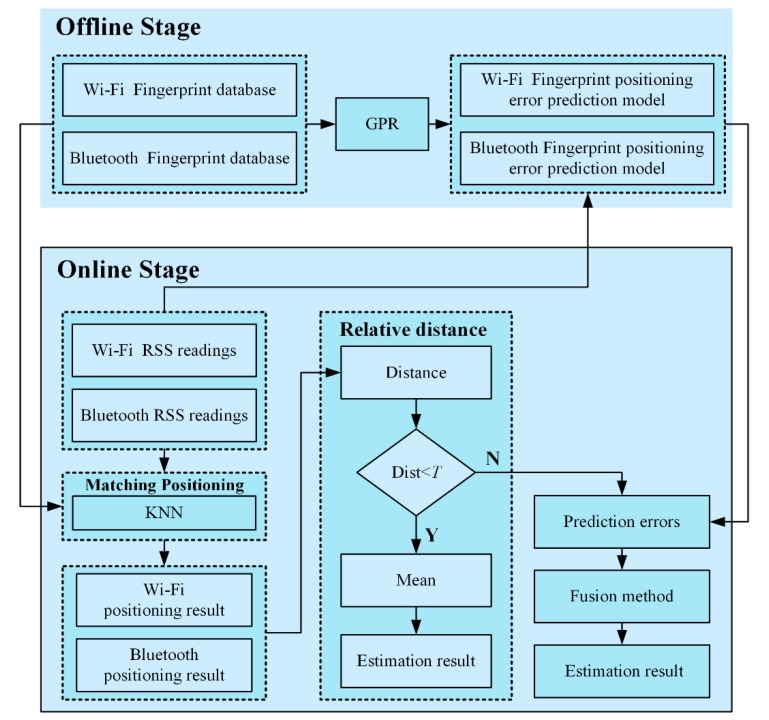
Flow diagram of an adaptive Bluetooth/Wi-Fi fingerprint positioning method based on Gaussian process regression (GPR) and relative distance (RD).

**Figure 3:**
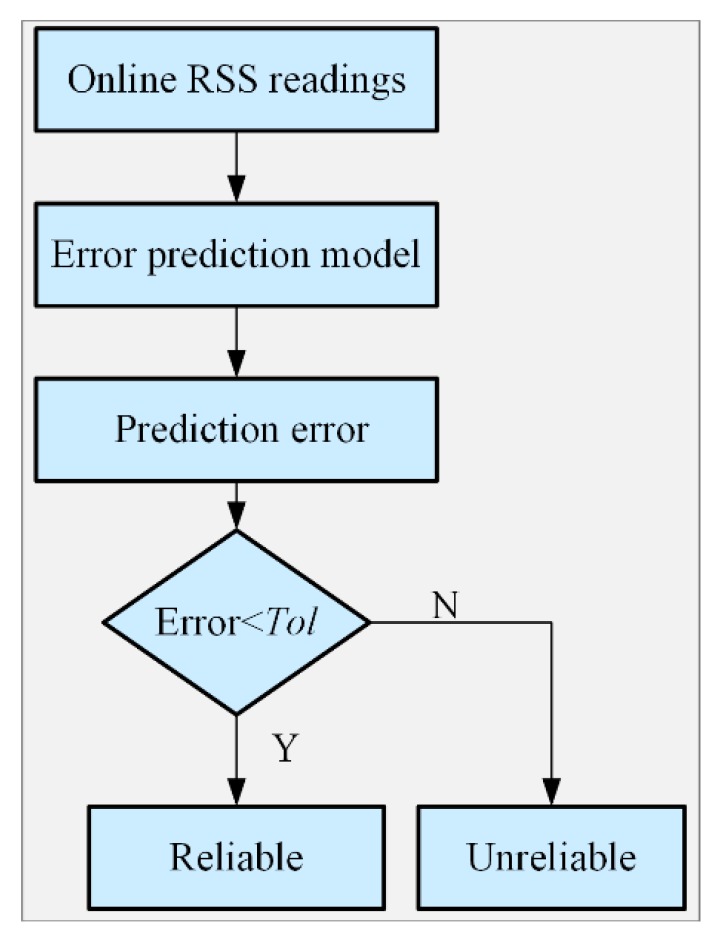
Flow diagram of the process of judging the credibility of the fingerprint positioning result.

As shown in Figure 2, in the offline phase, the Bluetooth and Wi-Fi RSS measurements were collected. The Bluetooth and Wi-Fi fingerprint databases were constructed by taking the RSS mean values. Subsequently, the fingerprint positioning error prediction models for BFP and WFP were produced by using training data and GPR. In the online stage, the online Bluetooth and Wi-Fi RSS readings were respectively matched with the Bluetooth and Wi-Fi fingerprint databases, respectively, for positioning with the KNN algorithm. The RD of BFPR and WFPR was calculated to determine whether they are both trusted. Both BFPR and WFPR are credible when the distance is less than the threshold *T*, and the mean of BFPR and WFPR is then regarded as the positioning estimation result. A distance between the two positioning results greater than *T* indicates an abnormal positioning result. The online RSS readings and positioning error prediction models were used to forecast the positioning errors of BFPR and WFPR, as shown in Figure 3. The positioning result is reliable when the prediction error is less than threshold *Tol*; otherwise, the positioning result is unreliable.

Finally, the fusion method was chosen according to the judgment result. When BFPR and WFPR are both believed, the mean of BFPR and WFPR should be taken as the positioning estimation result. BFPR is regarded as the position estimation result if only BFPR is believed. If only WFPR is believed, it will be chosen as the position estimation result. When BFPR and WFPR are both not trusted, the mean is still used as the position estimation result.

### 4.2. Threshold Selection

As indicated in Section 4.1, the training data for the positioning error prediction models come from the fingerprint databases. The reference fingerprints and their positioning errors are taken as the training data. It is necessary to determine firstly the thresholds *T* and *Tol* before judging whether BFPR and WFPR are trusted.

**Figure 4:**
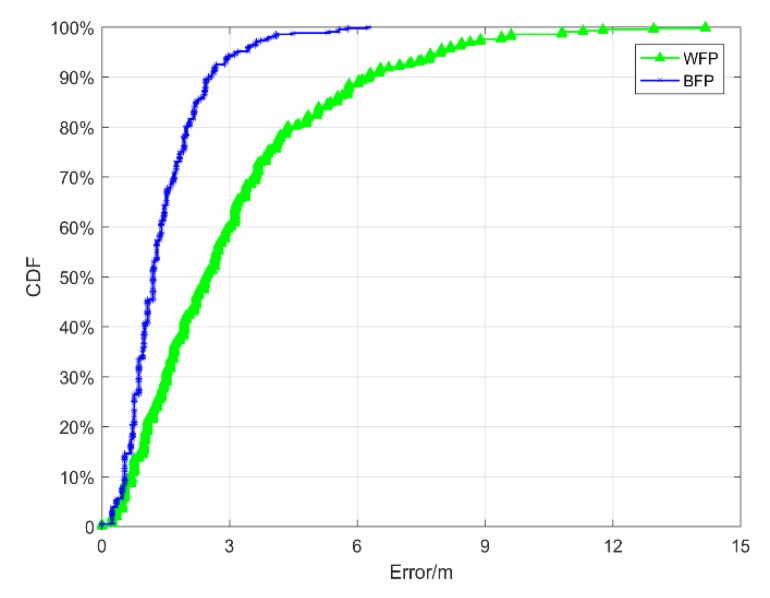
Cumulative distribution function (CDF) of positioning errors in the training data.

In order to select the threshold more accurately, this paper used the positioning errors in the training data to determine the threshold values *T* and *Tol.* Shown in Figure 4 is the cumulative distribution function (CDF) of positioning errors in the training data. Considering the cumulative probability of error, the *Tol* of BFPR and WFPR were chosen as the points where the cumulative probability of error was 0.7, which were 1.697 m and 3.608 m, respectively. When the prediction error of BFP was greater than 1.697 m, the positioning result was not trusted. When the prediction error of WFP was greater than 3.608 m, the positioning result was not credible. In addition, when the cumulative probability was 0.8, the error in WFP was 4.38 m. The error in BFP was 2.148 m when the probability was 0.95. Because both errors were within the acceptable range, and their corresponding cumulative probabilities were large enough, the sum was taken as the threshold value *T* (*T* = 7.509 m).

## 5. Experimental Environment and Analysis

### 5.1. Experimental Environment

**Figure 5:**
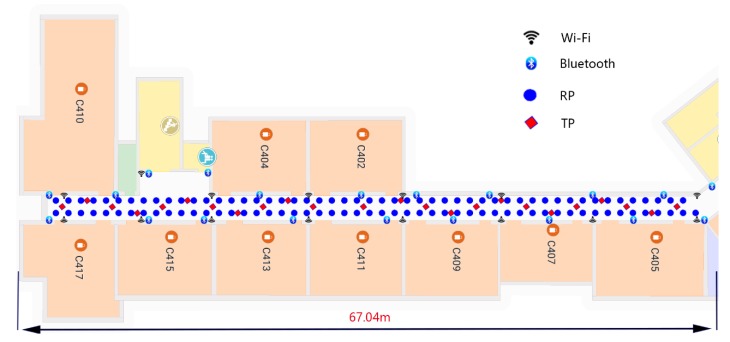
Experimental area.

The experimental environment is shown in Figure 5. There were 20 Bluetooth and 15 access points (APs) in the experimental area; in Figure 5, the circular points represent the RPs and the diamonds represent the test points (TPs). 

The experimenters stood still on each RP with a smartphone in hand and collected the Bluetooth and Wi-Fi RSS measurements at the same time. In order to avoid the influence of the experimenters’ body shielding on the experimental results, the RSS measurements in four directions were collected, and the acquisition time was 30 s in each direction with a collection frequency of 1 Hz. The RSS mean values of every direction were taken to construct the fingerprint database. There were four reference fingerprints on every RP. After the RP data collection was completed, the collection of TPs was carried out according to the same acquisition method as for the RPs. The experimenters collected the Bluetooth and Wi-Fi RSS measurements in four directions on the TP with an acquisition time of 10 s and acquisition frequency of 1 Hz. The RSS mean values were chosen as the test data. There were 412 reference fingerprints and 96 sets of test data used in the experiment.

### 5.2. Effect of Using Relative Distance Alone

In order to study the effect of using RD alone, in this section we used only the RD to realize adaptive Bluetooth/Wi-Fi fingerprint positioning. This was named as the adaptive Bluetooth/Wi-Fi fingerprint positioning method based on relative distance (ABWFPM_RD) in this work. Its positioning principle was to use the distance between BFPR and WFPR to determine whether the two positioning results are trusted. The distance being less than the threshold *T* shows that the two positioning results are to be believed, and the mean value is regarded as the positioning estimation result. If the distance is greater than *T*, there is an abnormal positioning result among the positioning results. The effect of BFP is better than that of WFP from Figure 4; thus the Bluetooth fingerprint positioning results were chosen as the position estimation results when the threshold was greater than *T*. The error mean (EM), maximum error (ME), and root-mean-square error (RMSE) were utilized for evaluating the positioning performances in this work. The RMSE is the standard deviation of positioning errors. The ME is the maximum value of the positioning errors. The EM is the mean of positioning errors, as shown in Equation (12):

(12) $$\mathrm{EM}=\frac{\sum_{i=1}^{S}e_{i}}{S}$$

where $e_{i}$ is the positioning error, and S is the number of positioning errors. The RMSE can be determined from the positioning errors and EM. The RMSE can be expressed as

(13) $$\mathrm{RMSE}=\sqrt{\frac{\sum_{i=1}^{S}{\left(e_{i}-\mathrm{EM}\right)}^{2}}{S}}$$

**Figure 6:**
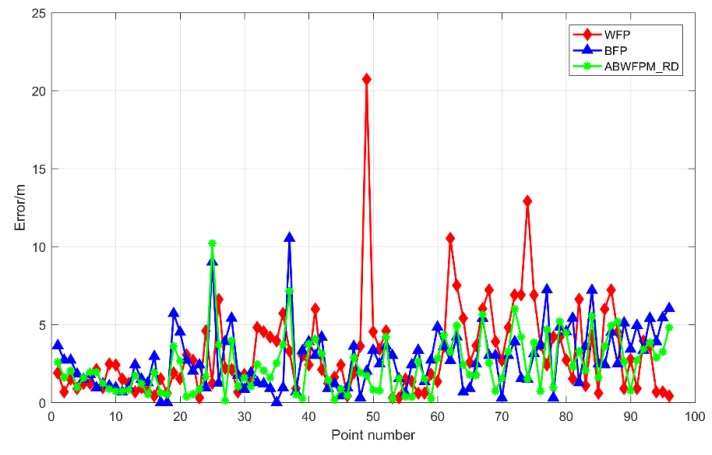
Positioning errors of adaptive Bluetooth/Wi-Fi fingerprint positioning method based on relative distance (ABWFPM_ RD), Wi-Fi fingerprint positioning (WFP), and Bluetooth fingerprint positioning (BFP).

Figure 6 shows the positioning errors of BFP, WFP, and ABWFPM_RD. The positioning effect of ABWFPM_ RD is better than those of BFP and WFP. Its number of large errors is less than those of BFP and WFP. The maximum positioning error of WFP was 20.72 m, while the maximum error of ABWFPM_ RD was 10.2 m, which indicates that ABWFPM_ RD can avoid the occurrence of larger errors. In addition, the EMs of WFP, BFP, and ABWFPM_ RD were 3.131 m, 2.771 m, and 2.372 m, respectively. Compared with WFP and BFP, the ABWFPM_ RD increased the positioning accuracy by 0.759 m and 0.399 m, respectively. The RMSEs of BFP, WFP, and ABWFPM_ RD were 2.978 m, 1.974 m, and 1.78 m, respectively. It can be seen that the positioning effect of ABWFPM_ RD obviously improved compared with WFP. However, when compared with BFP, the increases in the positioning accuracy and stability were quite limited, resulting in not obvious superiority.

### 5.3. Effect of Using Gaussian Process Regression Alone

This section analyzes the positioning effect of the adaptive Bluetooth/Wi-Fi fingerprint positioning method based on Gaussian process regression (ABWFPM_GPR). Its positioning principle is to use the positioning error prediction models to estimate the positioning errors of WFP and BFP. Subsequently, whether the fingerprint positioning results are trusted is determined according to the prediction error and threshold *Tol*. If only the BFPR is trusted, it should be chosen as the position estimation result. When only the WFPR is credible, it is chosen as the position estimation result. When both BFPR and WFPR are trusted or when neither are, the mean of BFPR and WFPR will be the positioning estimation result.

**Figure 7:**
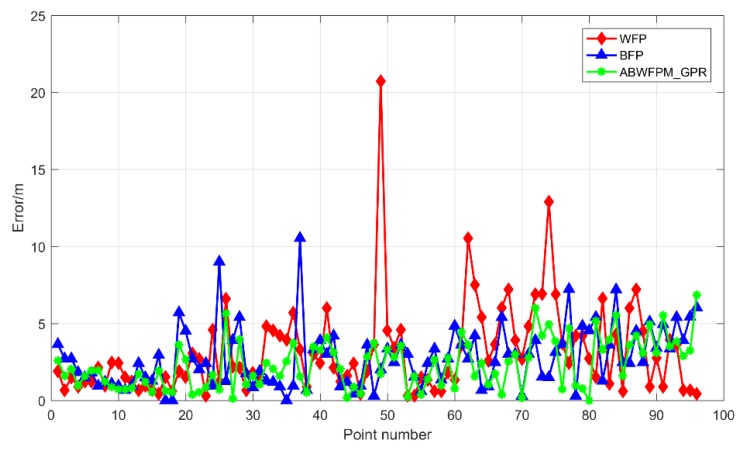
Positioning errors of ABWFPM_GPR, WFP, and BFP.

Figure 7 shows the positioning errors of ABWFPM_GPR, WFP, and BFP. It can be seen that ABWFPM_GPR has the best positioning effect. Its positioning accuracy and stability are significantly better than those of the other two methods. The EM of ABWFPM_GPR was 2.313 m, which is a decrease of 0.818 m when compared with WFP. The RMSE of ABWFPM_GPR was 1.58 m, which is a decrease of 1.398 m when compared with WFP. Compared with BFP, the EM and RMSE decreased by 0.458 m and 0.394 m, respectively. The improvement effect is bigger than that of ABWFPM_RD.

### 5.4. Adaptive Bluetooth/Wi-Fi Fingerprint Positioning Method Based on GPR and RD

#### 5.4.1. Comparison with BFP and WFP

RD can determine that both BFPR and WFPR are trusted or that neither are, but it is unable to determine which one is reliable. The positioning error prediction model can provide the prediction error, which can be used to judge whether a single positioning result is reliable or not and make up for the deficiency of RD.

**Figure 8:**
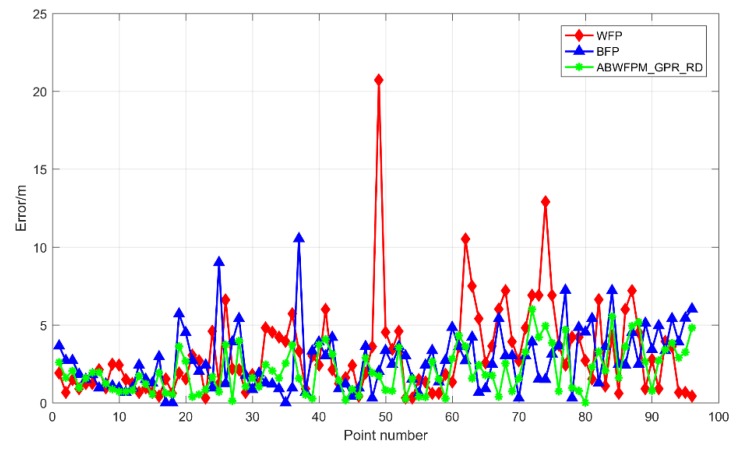
Positioning errors of ABWFPM_GPR_ RD, WFP, and BFP.

This section analyzes the adaptive Bluetooth/Wi-Fi fingerprint positioning method based on GPR and RD (ABWFPM_GPR_ RD). Figure 8 shows the positioning errors of ABWFPM_GPR_ RD, WFP, and BFP. The positioning effect of ABWFPM_GPR_ RD was optimal among ABWFPM_GPR_ RD, WFP, and BFP. The positioning accuracy and positioning effect were obviously superior to those of WFP and BFP.

**Figure 9:**
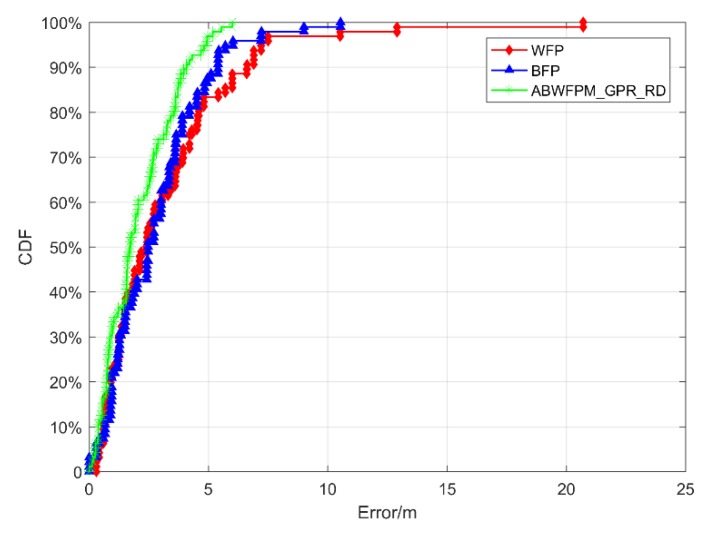
CDFs of positioning errors of BFP, WFP, and adaptive Bluetooth/Wi-Fi fingerprint positioning method based on GPR and RD (ABWFPM_GPR_ RD).

The CDFs of the positioning errors of BFP, WFP, and ABWFPM_GPR_RD are shown in Figure 9. The positioning accuracy of ABWFPM_GPR_RD was higher than that of BFP and WFP. The ME of ABWFPM_GPR_ RD was obviously smaller than that of BFP and WFP, and its positioning errors at different *y* values were also smaller than those of BFP and WFP. Therefore, the positioning effect of ABWFPM_GPR_ RD was better than that of BFP and WFP.

Table 1 shows the statistical results for the positioning errors of BFP, WFP, and ABWFPM_GPR_ RD. ABWFPM_GPR_RD had the best positioning effect. Compared with BFP and WFP, the positioning accuracy increased by 34.05% and 25.48% and the RMSE decreased by 1.529 m and 0.525 m, respectively. The positioning errors corresponding to the cumulative probabilities 50%, 70%, and 90% were much lower than those for BFP and WFP. The EM in the table represents the mean of positioning errors, and the ME represents the maximum value of positioning errors. The EM and ME of ABWFPM_GPR_RD were 2.06 m and 6.001 m, respectively. The positioning effect of ABWFPM_GPR_RD was obviously better than that of WFP and BFP.

#### 5.4.2. Comparison with ABWFPM_ RD and ABWFPM_GPR

**Figure 10:**
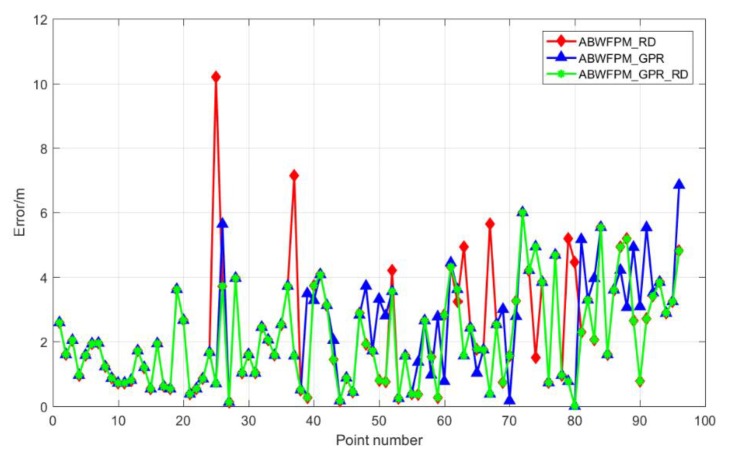
Positioning errors of ABWFPM_GPR_ RD, ABWFPM_ RD, and ABWFPM_GPR.

That ABWFPM_GPR_ RD was superior to BFP and WFP, as indicated in section 4.4, as it had higher positioning accuracy and stability. This section mainly compares the positioning effect of ABWFPM_GPR_ RD, ABWFPM_ RD, and ABWFPM_GPR, which is used to prove that RD and GPR used together are better than used alone. The experiment results—the positioning errors of ABWFPM_GPR_ RD, ABWFPM_ RD, and ABWFPM_GPR—are shown in Figure 10. The ABWFPM_GPR_ RD was implemented with an EM of 2.06 m and RMSE of 1.449 m. Its positioning accuracy and stability were the best among those of ABWFPM_GPR_ RD, ABWFPM_ RD, and ABWFPM_GPR.

**Figure 11:**
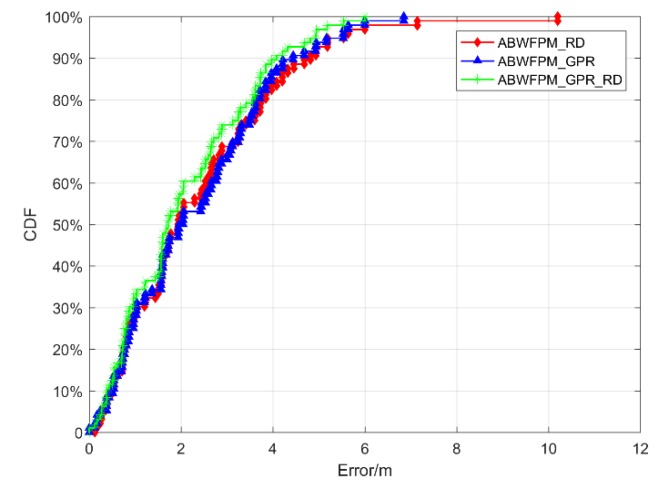
CDFs of the positioning errors of ABWFPM_GPR_ RD, ABWFPM_ RD, and ABWFPM_GPR.

The CDFs of the positioning errors of ABWFPM_RD, ABWFPM_ GPR, and ABWFPM_GPR_ RD are shown in Figure 11. When RD and GPR were used together, the positioning effect was obviously better than when used alone. The positioning accuracy of AFPM_GPR_ RD was higher than those of the other two positioning methods.

Table 2 shows the statistical results for the errors of ABWFPM_GPR_RD, ABWFPM_RD, and ABWFPM_GPR. The EM of ABWFPM_ RD was 2.372 m with an RMSE of 1.78 m. The EM and RMSE of ABWFPM_GPR were 2.312 m and 1.58 m, respectively. It was found that although ABWFPM_RD and ABWFPM_GPR showed greater improvement when compared with WFP, the improvement was rather limited when compared with BFP. However, the EM and RMSE of ABWFPM_GPR_ RD were 2.06 m and 1.449 m, respectively, which greatly improves the positioning accuracy and stability when compared with BFP and WFP. Its positioning effect also showed a certain improvement when compared with that of ABWFPM_RD and ABWFPM_GPR.

## 6. Conclusions

This paper proposed an adaptive Bluetooth/Wi-Fi fingerprint method based on Gaussian process regression and relative distance to realize the fusion of Bluetooth and Wi-Fi fingerprint positioning. This fusion showed a better positioning effect than Bluetooth or Wi-Fi fingerprint positioning alone. Its positioning accuracy and stability were also higher than those of an adaptive Bluetooth/Wi-Fi fingerprint method based on Gaussian process regression and an adaptive Bluetooth/Wi-Fi fingerprint method based on relative distance. It can make full use of the Bluetooth and Wi-Fi in indoor environments to realize indoor positioning with higher positioning accuracy and stability. However, the amount of matching computation required is larger than that required by Bluetooth and Wi-Fi fingerprint positioning; thus, in future research needs to investigate how to reduce the amount of fingerprint matching computation.

## Figures and Tables

**Table 1:** Statistical results of positioning errors (m).

Method	50%	70%	90%	ME	EM	RMSE
WFP	2.121	3.912	6.6	20.72	3.131	2.978
BFP	2.474	3.6	5.4	10.539	2.771	1.974
ABWFPM_GPR_RD	1.601	2.664	3.967	6.001	2.06	1.449

**Table 2:** Statistical results of positioning errors (m).

Method	50%	70%	90%	ME	EM	RMSE
FPMBW_ RD	1.772	3.122	4.68	10.201	2.372	1.78
FPMBW_GPR	1.935	3.122	4.215	6.85	2.312	1.58
AFPM_GPR_ RD	1.601	3.664	4.08	6.001	2.06	1.449
